# Art and science—Illustrator showcase: Adriana Savastano

**DOI:** 10.1002/ansa.202000106

**Published:** 2020-10-24

This issue's illustrator showcase highlights the artistic work of Adriana Savastano. We would like to thank Adriana for her work with Volmer et al., on their manuscript; Influence of core size and capping ligand of gold nanoparticles on the desorption/ionization efficiency of small biomolecules in AP‐SALDI‐MS. The full paper and Adriana's illustration can be found within the pages of this issue.^[^ ^1^, ^2^ ^]^

## 1 ILLUSTRATOR BIOGRAPHY

Adriana was born in Rome and studied biology at the University of Tor Vergata. Thereafter, she continued with a master's degree in molecular and cellular biology. During her master's studies, Adriana took part in the ERASMUS+ program, leading to a one‐year internship in Gottingen.

During her master's degree, Adriana became interested in neurodegenerative diseases together with the mechanisms of protein folding and aggregation leading her to the laboratory of Prof. Dr.Thomas Bayer, where she undertook her internship research investigating Amyloid beta plaques pathologies in mice models for Alzheimer's disease.

Following her master's and internship at Gottingen, Adriana pursued PhD research. Unable to reconcile the moral issue of animal studies but with a remaining passion to research Alzheimer's disease and neurodegeneration, Adriana focused on *in vitro* approaches. As a companion to this research, she followed her curiosity for structural biology studies, an interest piqued from her bachelor degree studies.

“I remember having a peculiar curiosity for Nuclear Magnetic Resonance (NMR) and its application for protein structural studies and I often said to myself; I need to find the courage to dive into this!"

Adriana's PhD adventure started in Gottingen in the laboratory of Prof. Dr. Markus Zweckstetter at the Max Planck for Biophysical Chemistry. Prof. Dr. Zweckstetter was (and still is) doing structural studies of proteins related to neurodegenerative diseases using NMR.

During the years of her PhD at the Max Planck for Biophysical Chemistry and the German Center for Neurodegenerative Diseases (in German DZNE), Adriana researched the Tau protein. This is a protein involved in the onset of Alzheimer's disease. Her research focused on understanding both its physiological and pathological functions by studying the protein's interaction with microtubules filaments and its aggregation into fibrils.

In order to undertake this research, Adriana advanced her knowledge of NMR theory, expanding on the experience gained from her more routine structural studies and developing further into multiple analytical techniques from circular dichroism, dynamic light scattering (DLS), optical microscopy, mainly using phase contrast and fluorescence microscopy, and many more in between.

“Now that my PhD adventure has come to an end, I continue research at Prof. Dr. Zweckstetter's this lab finalizing my current projects. I am very grateful for all the opportunities I had during these years!”

## 2 ILLUSTRATOR INTERVIEW

***When did you discover your passion for art?*** I always had an artistic side. When I was in school, my artistic personality was more predominant to the extent that no one would have ever thought I would become a scientist. I had put art aside during my studies at the university and at the beginning of my PhD, thinking I could only be either an artist or a scientist. I was wrong! During the second year of my PhD I stopped rejecting my artistic personality and started embracing it, merging it with science. It was the best decision I ever made

***What inspires your artwork?*** Almost everything! It can be a piece of music, a landscape view or a scene imagined after reading a book. And that is sometimes a problem, because I have way too many inspirations and too little time

***Would you briefly explain what your research group is studying?*** Our group studies the structure and dynamics of proteins related to neurodegenerative diseases. We study the physiological and pathological functions of protein involved in the onset of Alzheimer and Parkinson's disease using biophysical techniques, mainly NMR. We are also studying the process of liquid–liquid phase separation of proteins and its connection to neurodegeneration. Our main technique is NMR but we also use other biophysical techniques to study proteins dynamics.

***Why did you choose a career in spectroscopy?*** Since attending a course in structural biology with a focus on NMR, I became extremely curious toward what is beyond biology and toward the rules/phenomena that regulates macromolecular functioning. I was nevertheless always afraid to approach NMR, because of all the background in quantum mechanics and physic that is required to understand it.

***Of all your research projects, which one was your favorite and why?*** I have to admit, I enjoyed all of them. Of course, there were parts in each which I didn't like or found challenging (and for which I hated the project for a while). If I had to choose one, I would say the study of tau fibrils by solution and solid‐state NMR, because it is a project for which I learned many techniques and I feel like it was very complete.

***Who were the most influential people in your career?*** I met incredible scientists in my lab, they taught me all that I know and rescued me at the very beginning of my PhD. I would say they had a great impact in my path.

***What was the best advice you have received in your career?*** That each of us has his/her own experience and that we should never let us be influenced by others experiences.

***What are your favorite past‐times outside of science?*** I love playing the piano and the harp, drawing, painting, reading, and running …probably too many past‐time for the little time science leaves.

***What would you do if you had** **one** **‐year paid leave?*** I would not know what to choose, either travel or study music and art for a year in some conservatory or academy!

My gratitude to Dietrich is infinite! Working with him was my very first scientific illustration project and the first official opportunity. He trusted me completely and blindly! It would be awesome if other illustrators at "early stages" like me could have such amazing opportunities like I had with Dietrich!

Thanks to him I came into contact with a research very different to mine and which I would have never had the opportunity to know aboutotherwise! I would say we had an instant understanding of each other's expectations for this work and I really enjoyed talking with him. Especially I liked his view of science and of doing research: he is curious scientist, passionate, and striving for learning.

3

**FIGURE 1:**
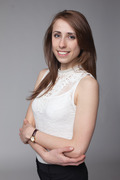
Adriana Savastano

**FIGURE 2:**
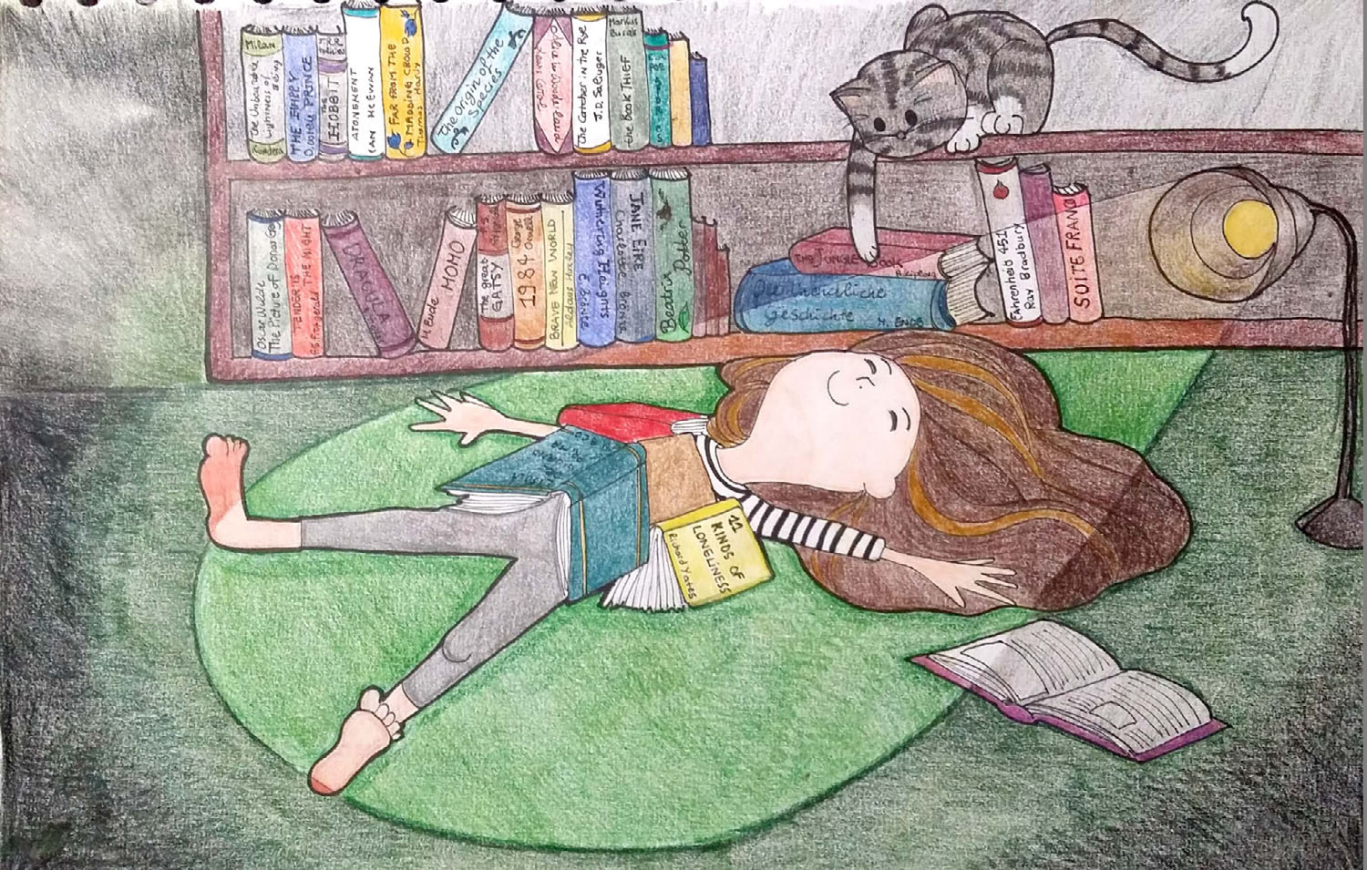
Portrait of a helpless reader and her cat, 2016

**FIGURE 3:**
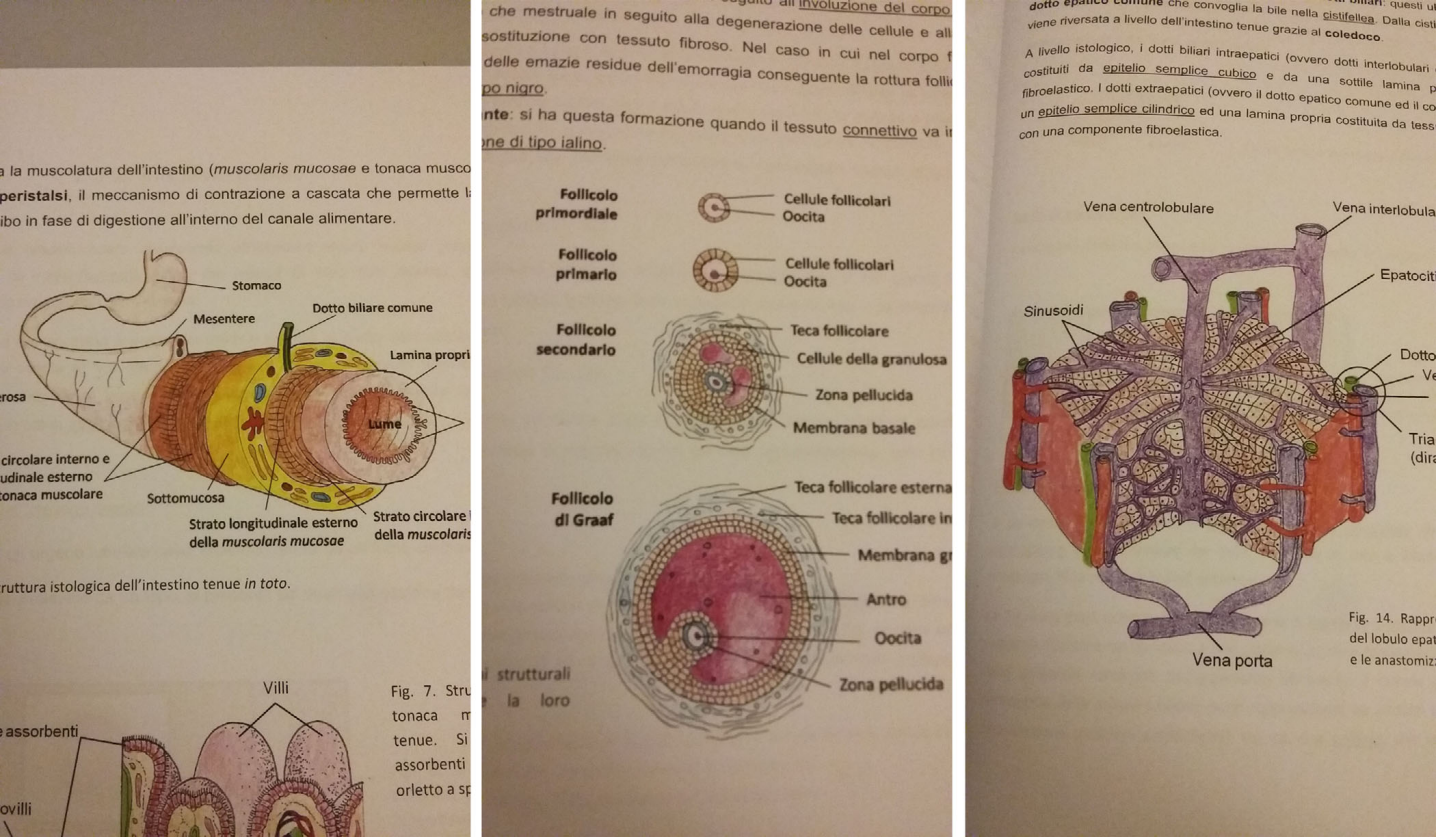
Scientific illustration for histology handouts for students of the faculty of biology of the University of Tor Vergata, 2011

**FIGURE 4:**
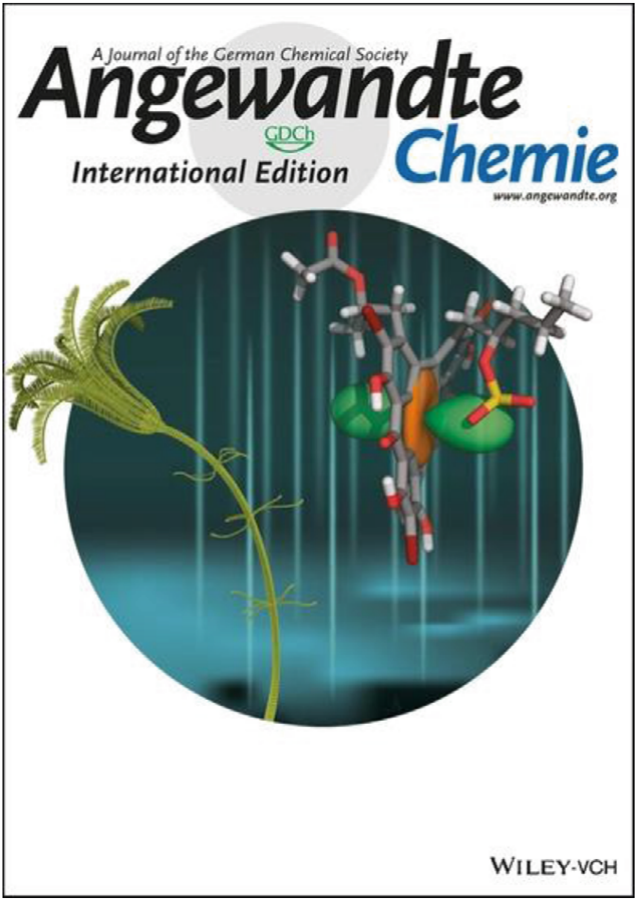
Angewandte Chemie cover feature by Adriana Savastano

**FIGURE 5:**
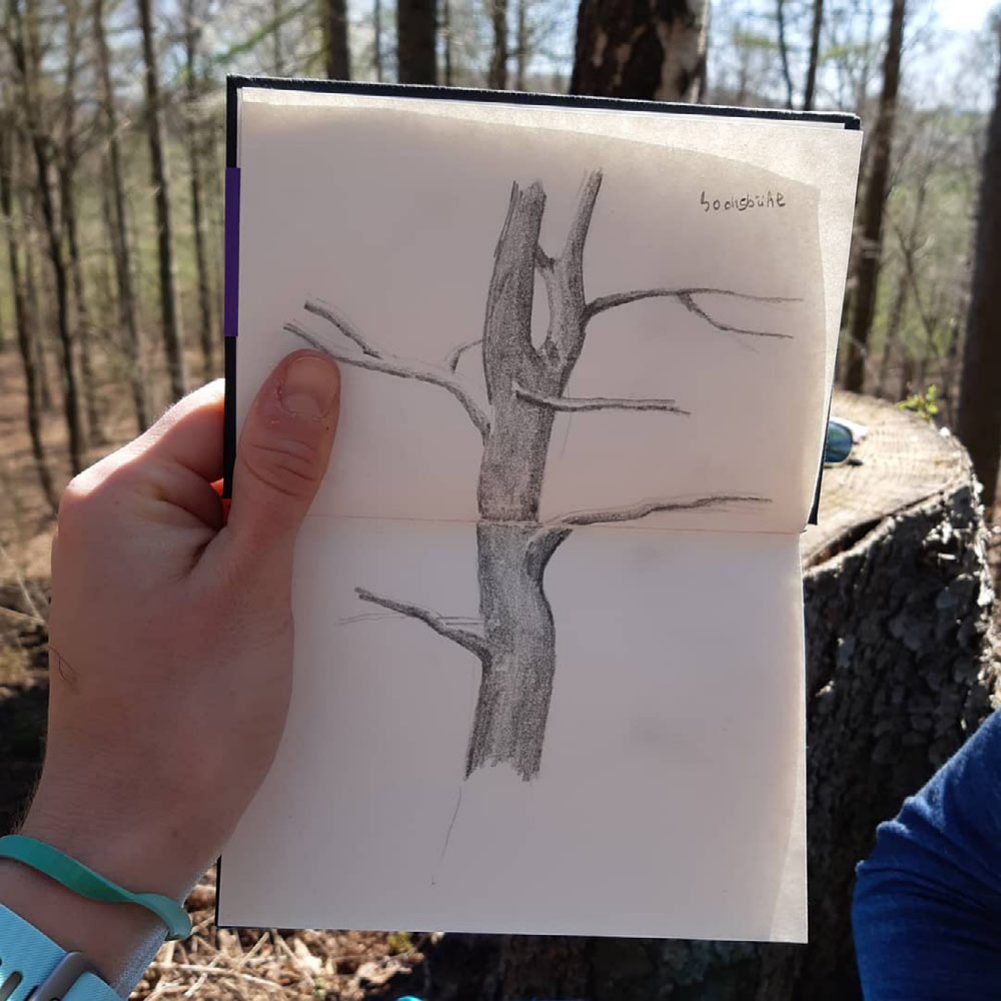
“Capturing a tree in the forest of Göttingen,” 2020

**FIGURE 6:**
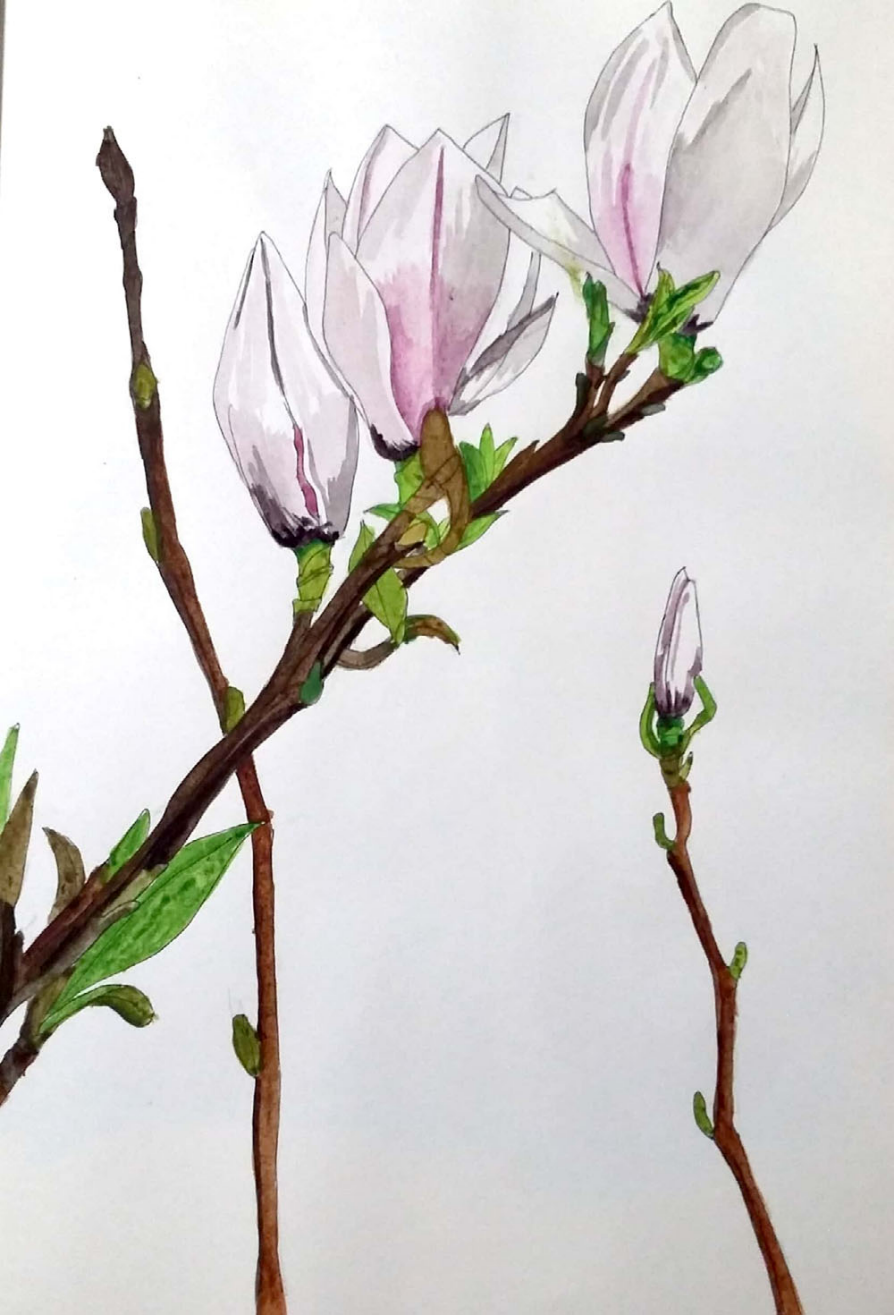
“Blooming Husum,” 2018

**FIGURE 7:**
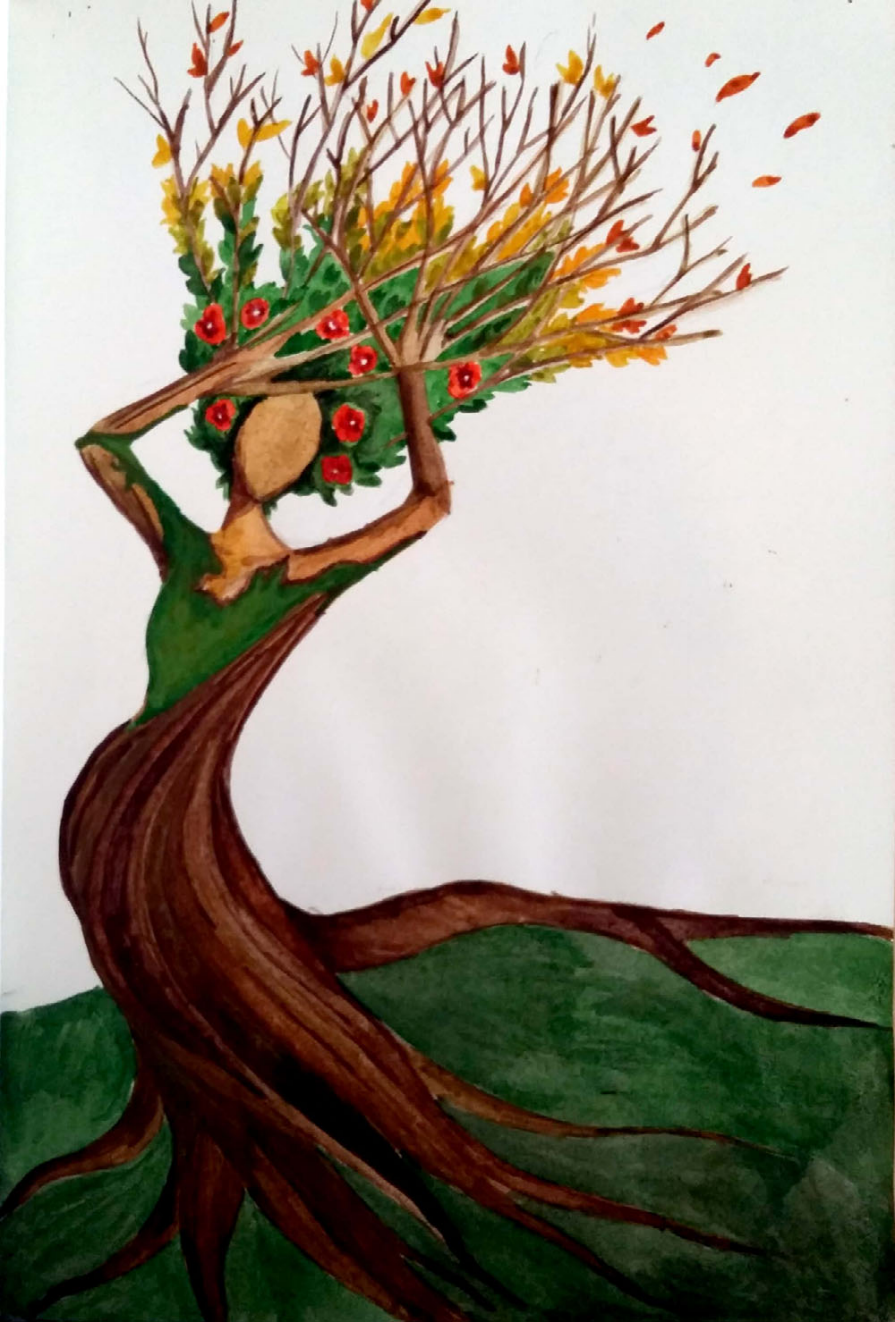
“Lost in the metamorphosis,” 2018

## References

[1] Liu Z , Zhang P , Pyttlik A , Kraus T , Volmer DA . Influence of core size and capping ligand of gold nanoparticles on the desorption/ionization efficiency of small biomolecules in AP‐SALDI‐MS. Anal Sci Adv. 2020;1:210‐220. 10.1002/ansa.202000002 PMC10989164 38716387

[2] Liu Z , Zhang P , Pyttlik A , Kraus T , Volmer DA . Influence of core size and capping ligand of gold nanoparticles on the desorption/ionization efficiency of small biomolecules in AP‐SALDI‐MS. Anal Sci Adv. 2020;1:209. 10.1002/ansa.202000900 PMC10989134 38716391

